# Meeting of the State Commissioners to Locate an Insane Asylum in the Eighth Judicial District

**Published:** 1869-07

**Authors:** 


### Meeting of the State Commissioners to locate an Insane Asylum in the Eighth Judicial District.

The Commissioners, Dr. John P. Gray of Utica, Dr. James P. White of Buffalo, Dr. William B. Gould of Lockport, Dr. Milan Baker of Warsaw, and Dr. Thomas D. Strong of Westfield, appointed by the Governor to locate an Asylum for the Insane in the Eighth Judicial District, held their first meeting at the residence of Dr. James P. White of this city. Invitations from different towns inviting the commissioners to visit them and examine proposed sites, as well as consider proposals for aid for the Asylum, if the location should be accepted, were received, read and laid before the commission for future consideration. It was resolved to visit all places extending such invitations, and to inspect the different sites proposed. Dr. J. P. Gray invited the commission to visit the Asylum in Utica, under his charge, as likely to afford better understanding of the importance and extent of the institution they were locating. This invitation was also accepted with much satisfaction as a most important preliminary to the undertaking.

The commission adjourned to meet the 28th day of September, 1869, at the same place.

We should not feel at liberty at present to speak concerning the place of location of this institution, had not the daily press, both in Buffalo and other places, opened the whole question without reserve. We understand that it is designed to accommodate the whole district to as great an extent as possible, and that the various towns which are in themselves very beautiful and attractive situations, must first consider whether they are so centrally and conveniently located as best to accommodate the whole district. The wishes of the few must certainly give place to the good of the many. This institution has been proposed by Buffalo men, and doubtless with every expectation that it would be located in Buffalo. Prof. James P. White and Senator Nichols have planned and thus far succeeded in this enterprise, and there can be no doubt that so far as originating and urging it upon the Legislature gives title, it is ours. But our strongest claims do not rest in this. It is proposed to establish an institution of great importance to Western New York and vicinity—an institution wholly in the interests of humanity, and not of towns and cities. Those who have planned it, and all who are interested in it, will readily concede that its location should be determined upon very broad principles. Buffalo, of course, expects the favor of choice, but, I believe, will be ready to listen attentively to claims which may be presented elsewhere, willing to promote the "greatest good of the greatest number." It is worth an effort in the various towns now proposing sites, for certainly these places would be great gainers in very many respects. The expenditure of nearly or quite a million of dollars in the first outlay and the subsequent supply, is certainly worth to our neighboring towns some effort; it would add to their population, business and wealth. Three thousand persons annually visit Utica, on business at the Asylum, making a longer or shorter stay, all adding to the business prosperity of the city. The location of a similar institution is a matter in which the wise men of all places will take the liveliest interest.

Buffalo will doubtless rely mainly on her location, believing that the inhabitants of the various towns offering sites will, without exception, be vastly better accommodated here than in any other place except their own. This simple fact is sufficient to determine the whole matter, almost regardless of the liberality of towns or cities desirous of securing it. The Eighth District cannot afford to gratify the natural pride and ambition of her thriving towns and villages by disadvantageous location of an institution of so much importance to all. What Buffalo will do in regard to offering a site, remains to be known. Upon a former occasion she offered generously and liberally; if called upon to do so, it is highly probable that the same wise liberality will prevail. Much might be said upon all the various points, but we propose to leave it with the Commission; composed as it is of men of learning, integrity, and wise discrimination, it cannot be left in truer, safer, or better hands.

